# The Relationship between Homocysteine Levels and Spontaneous Abortion in Iranian Women with Migraine

**Published:** 2017-08

**Authors:** Morteza NASIRI, Azam ARSANJANI SHIRAZI, Omid SADEGHI, Mahdieh BAGHERI BIDAKHAVIDI

**Affiliations:** 1. School of Paramedicine, Qom University of Medical Sciences, Qom, Iran; 2. School of Nursing and Midwifery, Dezful Islamic Azad University, Dezful, Iran; 3. School of Nutritional Sciences and Dietetics, Tehran University of Medical Sciences, Tehran, Iran; 4. School of Health, Kerman University of Medical Sciences, Kerman, Iran

## Dear Editor-in-Chief

Spontaneous abortion (SA) imposes great emotional and financial costs to young couples and society (1). Some recent investigations have shown hyperhomocysteinemia as a risk factor for SA; however, there is controversy in this regard (2, 3). Due to the importance of SA in pregnant women, regarding scare and conflicting data on relationship between homocysteine levels and SA, and since high homocysteine levels in patients with migraine can increase characteristics of migraine attacks such as its severity, frequency, and duration (4–6), we aimed to investigate the association between serum levels of homocysteine and SA among women with migraine.

This cross-sectional study was conducted on 76 women with migraine, aged 25–45 yr, in Khorshid and Emam Mosa Sadr clinics affiliated to Isfahan University of Medical Sciences, Isfahan, Iran, during 2014. Migraine was diagnosed by a neurologist according to International Headache Society (*IHS*) criteria.

Patients suffering from migraine in a long time with current diagnosis of migraine with aura and a one-year history of severe, recurrent attacks (1 to 8 attacks per month) were selected. Patients with chronic heart disease, previous stroke incidence, chronic renal failure, and with history of taking vitamin B supplements and history of abortion before migraine diagnosis were excluded from the study.

After getting approved by Ethical Committee of Isfahan University of Medical Sciences, and taking consent from all participants, we collected demographic characteristics (age, medical history, family history of migraine, taking of vitamin and mineral supplements and anti-migraine drugs in-take) and SA information (occurrence and time of SA) from each patient. In this study, SA was considered as pregnancy that ends before 20^th^ wk. Among 70 patients with migraine, 20 patients (28%) experienced SA. Mean homocysteine levels and the proportions of subjects with hyperhomocysteinemia were higher among patients with SA than those without (Table 1).

**Table 1:** Comparison of homocysteine levels and anthropometric measurements in patients with and without SA*

**Variable**	**Patients with SA**	**Patients without SA**	
	**Mean (SD) or N (%)**	**Mean (SD) or N (%)**	***P*-value**
Age (years)	38.17 ± 6.51	33.05 ± 6.59	0.03
BMI (kg/m^2^)†	27.63 ± 4.40	26.09 ± 4.85	0.24
WC (cm)††	86.51 ± 7.77	82.84 ± 8.95	0.12
Homocysteine (μm/l)	8.95 ± 2.61	7.32 ± 2.07	0.008
Hyperhomocysteinemia‡	8 (40%)	6 (12%)	0.012
Family history of migraine	13 (65%)	30 (60%)	0.45
Drug consumption‡‡	19 (95%)	44 (88%)	0.34

* Spontaneous abortion

† Body Mass Index

†† Waist Circumstance

‡ Considered as homocysteine concentration higher than 10μm/l

‡‡ long-term consumption of anti-migraine drugs such as corticosteroids and analgesics drugs

Multivariable odds ratio and 95% confidence intervals for the association between homocysteine levels and SA are presented in Table 2. In crude model, there was a significant positive relationship between homocysteine levels and SA (*P*=0.006). This association remained significant even after adjustment for potential confounding variables such as BMI, WC, family history of migraine and long-term anti-migraine drugs in-take (*P*=0.034).

**Table 2:** Results of logistic regression for association between homocysteine levels and SA*

**Variable**	**Total**		**Age < 35**		**Age ≥ 35**	
	OR (95% CI)	*P*	OR (95% CI)	*P*	OR (95% CI)	*P*
Crude	1.41 (1.10–1.80)	0.006	0.91 (0.57–1.47)	0.70	1.58 (1.13–2.19)	0.006
Model 1	1.43 (1.09–1.88)	0.009	1.16 (0.59–2.32)	0.67	1.83 (1.21–2.76)	0.004
Model 2	1.43 (1.08–1.90)	0.012	1.14 (0.57–2.27)	0.70	1.80 (1.19–2.74)	0.006
Model 3	1.35 (1.02–1.79)	0.034	1.16 (0.59–2.27)	0.67	1.72 (1.14–2.58)	0.009

* Spontaneous abortion

Model 1: Adjusted for BMI and WC

Model 2: Additionally adjusted for family history of migraine

Model 3: Further controlled for long-term consumption of anti-migraine drugs such as corticosteroids and analgesics drugs

Stratified analysis by age revealed a significant positive association between homocysteine levels and SA among patients 35 yr and older (*P*=0.006). This relationship was significant even after adjustment for potential confounders (*P*=0.009). No significant relationship was found between homocysteine levels and SA among patients less than 35 yr.

In spite of several studies, which assess this relationship between homocysteine levels and SA in other populations, this study examined this association among migraine patients. In consistent with our results, homocysteine level was associated with increased risk of SA (7). In addition, *w*omen with unexplained recurrent SA had high levels of serum homocysteine (6).

The high homocysteine levels are positively associated with SA among patients over 35 yr, without any significant association in patients below 35 yr old. A large number of studies have found a significant correlation between age and pregnancy loss. Therefore, high homocysteine levels in the older age range may increase the incidence of SA.
